# Complete Genome Sequence of *Staphylococcus aureus* Siphovirus Phage JS01

**DOI:** 10.1128/genomeA.00797-13

**Published:** 2013-11-14

**Authors:** Hongying Jia, Qinqin Bai, Yongchun Yang, Huochun Yao

**Affiliations:** Key Lab Animal Bacteriology, Ministry of Agriculture, College of Veterinary Medicine, Nanjing Agricultural University, Nanjing, Jiang Su, China

## Abstract

*Staphylococcus aureus* is the most prevalent and economically significant pathogen causing bovine mastitis. We isolated and characterized one staphylophage from the milk of mastitis-affected cattle and sequenced its genome. Transmission electron microscopy (TEM) observation shows that it belongs to the family *Siphovirus*. We announce here its complete genome sequence and report major findings from the genomic analysis.

## GENOME ANNOUNCEMENT

*Staphylococcus aureus* is a Gram-positive bacterium and the causative agent of several human diseases, including skin infections, endocarditis, and food poisoning (1). It is also a major causative bacterium of bovine mastitis, which results in serious economic losses (2). Phages are potential agents for treatment of antibiotic-resistant bacteria, as they lyse specific bacteria. Much evidence from animal model studies conducted in Western countries since 1980 supports the effectiveness of phage therapy against bacterial infectious diseases (3–5). We isolated and characterized an *S. aureus* phage, designated JS01, from the milk of mastitis-affected cattle. Transmission electron microscopy (TEM) showed that the phage has a long noncontractile tail and an icosahedral head and therefore belongs to the family *Siphovirus* in the order *Caudovirales*. Here, we report the complete genome sequence and organization of the novel bacteriophage JS01, which infects *S. aureus*.

The phage DNA was extracted using the alkaline lysis method with some modifications (6). It was sequenced by using the GS-FLX DNA library preparation kit (Roche Applied Science, USA), amplified by emulsion PCR (emPCR), and sequenced on a GS-FLX (454 Life Sciences, USA). The 454 reads were assembled with Newbler (version 2.0) (Roche, USA) using the default assembly parameters. The putative opening reading frames (ORFs) were predicted using Glimmer 3.0 (http://www.ncbi.nlm.nih.gov/projects/gorf/) and Prodigal (http://compbio.ornl.gov/prodigal/). BLASTn and BLASTp (http://www.ncbi.nlm.nih.gov/blast/Blast.cgi) were used to search the homologous genes and deduced amino acid sequences. tRNAs were predicted using the tRNAscan-SE 1.23 software (http://lowelab.ucsc.edu/tRNAscan-SE/).

The JS01 genome is 43,458 bp long, with a G+C content of 33.32%, 66 ORFs, and no tRNAs. Of the predicted ORFs, 46.96% are hypothetical or unknown proteins due to insufficient database information about the functional genes of *S. aureus* phage genomes. The gene-coding potential of the global genome is 88.93%. The structural and morphogenesis proteins include tail length tape-measure protein, phage minor structural protein, capsid protein, phage head-tail adaptor, and phage major tail protein. Many nonstructural proteins are thought to be involved in DNA replication and regulation, packaging, lysis, and lysogeny, including staphylococcal complement inhibitor, integrase, repressor homolog, phage anti-repressor protein, phage-related DNA recombination protein, single-stranded DNA-binding protein, phage regulatory protein, crossover junction endodeoxyribonuclease RusA superfamily protein, integrase regulator RinB, small subunit of phage terminase, large subunit of phage terminase, portal protein, and prohead protease. One toxin gene (staphylokinase) was found in the genome. Staphylokinase has a high fibrinolytic activity (7). Interestingly, a comparative genome analysis revealed that the *S. aureus* phage JS01 genome is 99% identical to that of *S. aureus* strain MSSA476 (GenBank accession no. BX571857.1), with 55% coverage, and it is 99% identical and has 71% coverage to *Staphylococcus* phage PVL (Genbank accession no. AB009866.2), which was isolated from mitomycin C-induced *S. aureus* V8 (ATCC 49775) (8). These suggest that the phage genome has a high identity with the *Staphylococcus* and other *Staphylococcus* phages. Studies investigating the complete genome of phage JS01 would provide novel information about *S. aureus* and the new methods of treating clinical infectious diseases caused by *S. aureus*, especially multidrug-resistant strains.

### Nucleotide sequence accession number.

The complete genome sequence of *Staphylococcus aureus* JS01 was deposited in GenBank under the accession no. KC342645.
